# DBSCAN applied to EHRs data from patients with glioblastoma clusters patients based on cytosolic Hsp70 protein, sex, and brain subventricular zone

**DOI:** 10.1186/s13040-026-00549-x

**Published:** 2026-03-27

**Authors:** Davide Chicco, Srinjoy Dora, Luca Oneto

**Affiliations:** 1 Università di Milano-Bicocca, Milan, Italy; 2 University of Toronto, Toronto, ON Canada; 3 Tbilisi State Medical University, Tbilisi, Georgia; 4 Universitá di Genova, Genoa, Italy

**Keywords:** Clustering, Unsupervised machine learning, Machine learning, Glioblastoma, Electronic health records, EHRs

## Abstract

Glioblastoma is an aggressive brain cancer that kills approximately one hundred thousand people worldwide every year. Unfortunately, treatment and therapy for patients with this disease are complicated and have limited efficacy in improving individuals’ chances of survival. Electronic health records (EHRs) contain patient information collected routinely at hospitals through medical visits and laboratory tests, providing an interesting source of data for computational analyses. Clustering is an area of unsupervised machine learning where an algorithm partitions data according to certain statistical properties or rules, thereby identifying hidden patterns and correlations that would otherwise be difficult to notice. In this study, we applied several clustering techniques to three open datasets (Munich2019, Tainan2020, and Utrecht2019) derived from electronic health records, which included clinical, genetic, and administrative features of patients diagnosed with glioblastoma, considering two possible clusters. We evaluated our clustering results with the Density-Based Clustering Validation (DBCV) index, a relatively new score capable of accurately assessing both convex-shaped and concave-shaped clusters. Among the methods tested, Density-based Spatial Clustering of Applications with Noise (DBSCAN) yielded the best results across all three datasets. We then analyzed the features of the clusters identified by DBSCAN and found that cytosolic Hsp70 protein in the Munich2019 dataset, sex in the Tainan2020 dataset, and brain subventricular zone in the Utrecht2019 resulted significantly capable to distinguish the two clusters.

## Introduction

Glioblastoma is an aggressive type of brain cancer that originates from glial cells, which are supportive cells in the nervous system  [1]. Glioblastomas are characterized by rapid growth, and they can infiltrate surrounding brain tissue, making them difficult to treat. Symptoms may include headaches, seizures, cognitive changes, and neurological deficits, depending on the tumor’s location in the brain  [1]. Data derived from electronic health records (EHRs) of patients having this cancer type can be used for computational analyses that, in turn, can lead to significant discoveries in medical sciences. In the past, we used supervised machine learning models on EHRs data of three open glioblastoma curated datasets to infer the most prognostic clinical factors  [2]. In the present study, we reuse the same three open curated datasets for an unsupervised analysis, aimed at identifying clusters of patients which might have a particular medical relevance. In the past, several studies employed computational clustering techniques to analyze glioblastoma data, but mainly within bioinformatics and computational biology  [3–10]. Supervised machine learning was employed in several projects involving data of patients with glioblastoma  [11], but we could not find any study regarding unsupervised analyses of data of patients with this disease. To the best of our knowledge, no studies currently exist in the scientific literature applying clustering or unsupervised machine learning to EHRs data from patients with glioblastoma. We fill this gap by presenting this study aimed at detecting groups of patients having particular medical meaning. Our main goal is to discover clusters that have a clinical-pathological meaning, by answering the following questions: can clustering methods identify relevant stable groups of patients with glioblastoma in these three independent datasets? If yes, do these groups have a clinical-biological meaning?

Our results confirmed that density-based clustering is an effective tool for this scientific goal and that three particular clinical variables were so relevant that they could divide the two clusters found in a precise way.

We organize the rest of this article as follows. After this Introduction, we describe the three analyzed datasets in section “Datasets”, and explain the algorithm we used in section “Methods”. We report and describe the results we obtained in section “Results” and then discuss them with the limitations and future developments of this study in section “Discussion and conclusions”.

## Datasets

We analyzed three open datasets derived from electronic health records (EHRs) of patients diagnosed with glioblastoma, that we called Munich2019 dataset  [12, 13], Tainan2020 dataset  [14], and Utrecht2019  [15]. A description of the clinical features of these datasets can be found in our previous study  [2].

The Tainan2020 dataset was collected in a medical centre in Tainan (Taiwan) from 2005 to 2018, and contains data from 84 patients, each having 9 clinical features. This dataset has no missing data (Table 1). The Utrecht2019 dataset contains data of patients diagnosed with supratentorial glioblastoma between 2005 and 2013 in the Netherlands and consists of data from 647 patients and 7 variables (Table 1). Some values are missing for a few features: KPS 0.62%, adjuvant treatment 0.77%, and SVZ contact 5.56% (Table 1). The Munich2019 dataset is made of data from 60 patients, each having 7 features as well, and was collected in Germany before 2012 (Table 1). Some values are absent from this dataset, too: radiation volume for 32%, adjuvant temozolomide (TMZ) treatment for 25%, and progress free survival (PFS) for 5.95% (Table 1).

**Table 1:** Quantitative description and references of the three analyzed datasets

dataset	# patients	# features	% missing	references
Munich2019	60	7		[12, 13]
Tainan2020	84	14	$\sim4\%$	[14]
Utrecht2019	647	7	$\sim1\%$	[15]

The three raw datasets are openly available on Figshare.com under the CC BY 4.0 license

These three anonymous datasets were released publicly online on Figshare under a Creative Commons Attribution 4.0 International (CC BY 4.0) license by the original dataset curators, following the FAIR principles  [16]. We then published our cleaned datasets within an R package on CRAN  [17].

More information about these datasets can be found in a previous machine learning study  [2] and in the original datasets’ articles  [12–15].

Regarding representativeness, these datasets originate from three different countries and contain data from approximately equal numbers of women and men, with ages ranging from 20 to 80 years. Age and sex are common variables across all three cohorts, while survival data is shared only between the Utrecht2019 and Munich2019 datasets. This diversity makes the three datasets sufficiently representative for drawing global conclusions from our findings.

In terms of dataset size, only the Utrecht2019 dataset with data profiles from 647 patients provides a sufficiently large number of data points for robust cluster analysis. The Munich2019 dataset (60 patients) and the Tainan2020 dataset (84 patients), however, have smaller sample sizes, which makes them more challenging to analyze. Nonetheless, given the rarity of glioblastoma medical record datasets, we believe that our cluster analysis of these three datasets can still reveal valuable insights and findings for the scientific community.

## Methods

### Preprocessing

Before applying unsupervised clustering algorithms to the three datasets, we performed some data preprocessing steps. There, we imputed missing data by replacing absent data elements with mean values for numerical features and the most frequent values for ordinal and categorical variables. We transformed all the categorical variables into binary numerical features through the one-hot encoding strategy  [18]. We normalized all variables using a standard scaler to the zero-one range and treated ordinal variables as numerical values.

### Algorithms

After preprocessing, we applied several unsupervised clustering methods to the three medical datasets of patients with glioblastoma, and DBSCAN  [19] obtained the best results, measured as density-based clustering validation (DBCV index)  [20, 21]. We followed the example of another similar study of ours on neuroblastoma  [22].

We selected ten of the most common clustering methods employed in the health informatics literature, for which an open source Python implementation is available  [23]: DBSCAN  [19], $k$-Means  [24], Spectral Clustering  [25], Agglomerative Clustering  [26], BIRCH  [27], Gaussian Mixture  [28], Affinity Propagation  [29], MeanShift  [30], OPTICS  [31], and HDBSCAN  [32].

We performed a data-driven grid search for hyperparameter tuning by trying several values and then picking the ones which generated the highest DBCV index. For DBSCAN, we tried all the minimal samples in the $(2,4,8,16,32,60)$ interval and all the epsilon values logarithmically spaced between $10^{-4}$ and $10^{+3}$, with the Euclidean distance.

### Result assessment

The DBCV index is a density-based version of the Silhouette coefficient  [33], which is a common metric employed for clustering internal assessment. The original Silhouette coefficient can work well when assessing convex clusters, but can mislead when employed to evaluate concave or nested clusters. The DBCV index solves this problem by considering the density of the clusters in its formula, and generates a score in the $[-1, +1]$, where $-1$ means disastrous clustering, 0 means a clustering no better than random chance, and $+1$ means perfect clustering. Differently from other clustering methods such as $k$-means or hierarchical clustering, DBSCAN assigns data points not only to real clusters but also to a noise cluster. This noise cluster consists of data points that do not belong to any real cluster, according to the DBSCAN partitioning.

We decided to consider only results generated through two clusters because it is a medically appropriate number of clusters used in several glioblastoma biomedical informatics studies  [34–36].

**Fig. 1:**
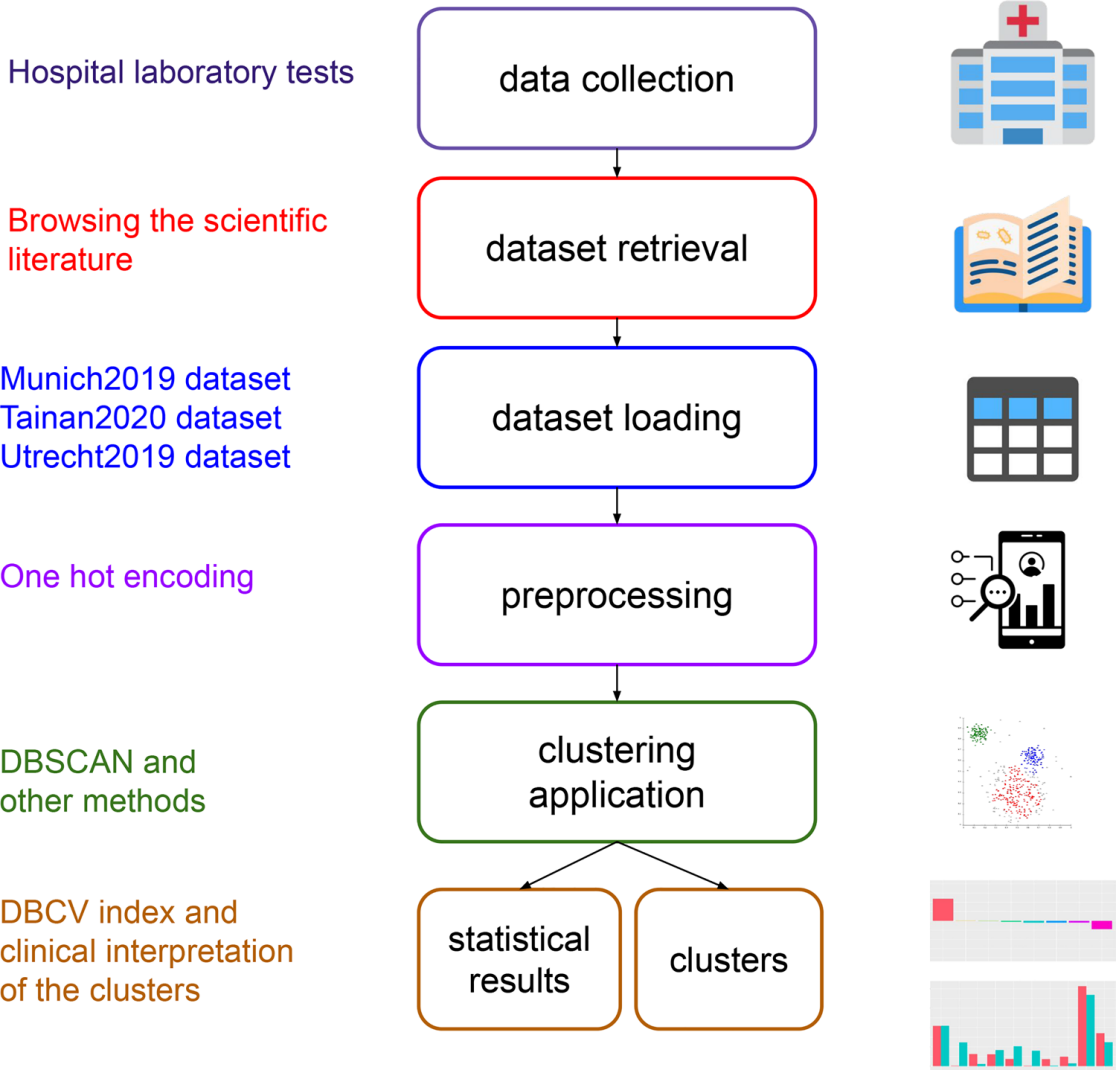
Schematic representation of our study process. The data collection phase was conducted by the original datasets curators in their hospitals  [12–15]. We conducted the dataset retrieval via scientific literature search engines. The steps from dataset loading to statistical results and clusters refer to the computational analysis presented in this study. All the illustration images were released online publicly under a Creative Commons license: the hospital icon from IconScout.com, the book icon from IconScout.com, the table icon from Wikimedia Commons, the barchart icon from IconScout.com, the clusters image from Wikimedia Commons. We adapted this image from Figure 1 of  [22], that was released under the creative Commons attribution 4.0 International (cc BY 4.0) license

We represent the computational pipeline of our project in Figure 1.

### Software packages

For this study we employed the following main Python 3 packages: NumPy  [37], pandas  [38], scikit-learn  [39], and SciPy  [40]. We decided to use Python because it is an open-source programming language, which facilitates the reproducibility of the computational analysis  [41].

## Results

### Clustering results

We applied several clustering algorithms and DBSCAN obtained the best results. We reported the results obtained by the other algorithms in Table 2.

**Table 2:** Results generated by the other clustering algorithms on the three datasets analyzed, with the optimized hyperparameters

DBCV	# cluster 0	# cluster 1	# noise cluster	Method	Hyperparameters
**Munich 2019**					
+0.349	56	4	0	Spectral Clustering	gamma: 0.889, n components: 6
+0.291	58	2	0	BIRCH	branching factor: 2, threshold: 0.889
+0.280	57	2	1	HDBSCAN	minimal samples: 2
−0.061	37	23	0	Gaussian Mixture	covariance type: diag
−0.062	34	26	0	$k$-Means	
−0.074	35	25	0	Ward	linkage: ward, metric: euclidean
−0.080	49	7	4	OPTICS	min samples: 16; xi: 0.02
**Utrecht 2019**					
+0.0112	645	2	0	Spectral Clustering	gamma: 16.037, n components: 2
+0.0112	645	2	0	Agglomerative Clustering	linkage: average, metric: cosine
−0.001	507	140	0	BIRCH	branching factor: 8 threshold: 0.258
−0.002	223	424	0	Gaussian Mixture	covariance type: tied
−0.004	492	155	0	Ward	linkage: ward metric: euclidean
**Tainan 2020**					
+0.862	17	16	51	OPTICS	min samples: 2, xi: 0.01
+0.615	19	6	59	HDBSCAN	min cluster size: 2
+0.137	78	6	0	Spectral Clustering	gamma: 7.017, n components: 5
+0.084	82	2	0	BIRCH	branching factor: 2 threshold: 0.889
+0.047	76	8	0	Agglomerative Clustering	linkage: average, metric: cosine
+0.042	52	32	0	$k$-Means	
+0.042	52	32	0	Gaussian Mixture	covariance type: full

OPTICS and HDBSCAN did not produce sufficient results with 2 clusters for the Utrecht 2019 dataset

**Fig. 2:**
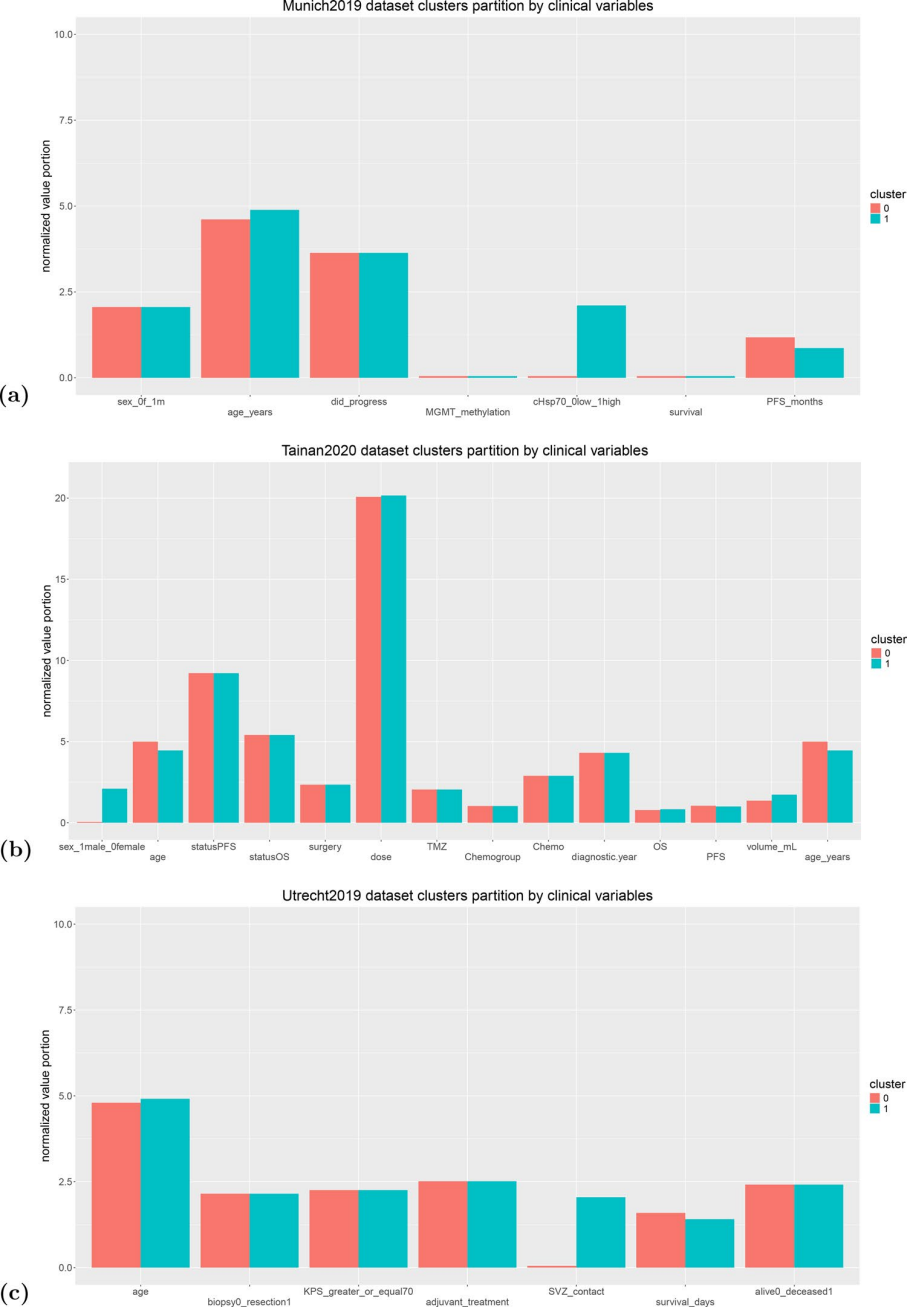
Partition of the clinical features among the two clusters identified by DBSCAN. Representation of the normalized values of the clinical variables of teach dataset in the subset of patients of the 0 cluster (red bars) and in the subset of patients of the 1 cluster (green bars). (**a**) top image: Munich2019 dataset. (**b**) mid image: Tainan2020 dataset. (**c**) bottom image: Utrecht2019 dataset. We listed the meaning of the clinical variables in  [2]

DBSCAN outperformed the other clustering algorithms we employed by obtaining a DBCV index of +0.963 in the Munich2019 dataset, of +0.923 in the Tainan2020 dataset, and of +0.961 in the Utrecht2019 dataset, respectively (Table 3 and Figure 2). On the three analyzed datasets, DBSCAN assigned to real clusters around 28% of patients in the Munich2019 dataset, around 31% of patients in the Tainan2020 dataset, and around 24% of patients in the Utrecht2019 dataset  [20] (Figure 2).

**Table 3:** Results obtained by DBSCAN

Dataset	DBCV	# cluster 0	# cluster 1	# noise cluster	# patients	cluster 0 & 1
Munich2019	+0.963	7	10	43	60	28.33%
Tainan2020	+0.923	13	13	58	84	30.95%
Utrecht2019	+0.961	74	80	493	647	23.80%

# cluster 0: number of patients assigned by DBSCAN to the first cluster. # cluster 1: number of patients assigned by DBSCAN to the second cluster. cluster 0 & 1: percentage of patients assigned by DBSCAN to the first or the second cluster, and not assigned to the noise cluster. # patients: number of patients. Hyperparameters: 2 clusters for DBSCAN in each test and the following epsilon values and minimal points Munich2019 dataset epsilon: 0.232, minimal samples: 4. Tainan2020 dataset epsilon: 0.286, minimal samples: 4. Utrecht2019 dataset epsilon: 0.429, minimal samples: 64. The DBCV index ranges from −1 (worst outcome) to + 1 (best outcome)

Regarding outliers, we notice that DBSCAN inserted a huge number of patients in the −1 noise cluster: around 72% for Munich2019, around 69% for Tainan2020, and around 76% for Utrecht2019 (Table 2), for an average of 72%. We believe this outcome is due to the extreme heterogeneity of the variables of these three datasets: seeing so many diverse data types (integer numbers, real numbers, ordinal classes, and binary categories) from the three independent datasets, DBSCAN consider several data elements as outliers, and treats them accordingly.

We observed that DBSCAN assigned a large number of patients to the −1 noise cluster: approximately 72% for Munich2019, around 69% for Tainan2020, and about 76% for Utrecht2019 (Table 2), resulting in an average of 72%. We believe this outcome is due to the extreme heterogeneity of the variables across these three independent datasets. Given the presence of many diverse data types (integer values, real numbers, ordinal classes, and binary categories) from three independent sources, DBSCAN probably identified many data points as outliers and treated them accordingly.

Even though the two clusters identified by DBSCAN are small, we believe they may hold relevant medical significance. Another way to approach our project is to examine whether DBSCAN can identify some patients as non-outliers, and then investigate if these patient groups share any meaningful clinical characteristics.

A large noise cluster can result from data heterogeneity or data sparsity, meaning that many data points are excluded by the algorithm’s threshold. In our results, the typical heterogeneity of data derived from EHRs  [42] is the primary cause of the large noise clusters observed in the three datasets considered. EHRs data, in fact, contain variables of several types: real-valued, binary categorical, and ordinal. This heterogeneity complicates the analysis of EHRs data and consequently influences the clustering results.

### Medical results

We then analyzed the content of the two clusters detected by DBSCAN in the three datasets, and observed the proportions of their clinical variables. Here we investigate what other biomedical studies say about the biomedical features resulted more relevant from the clustering analysis.

In the Munich2019 dataset, DBSCAN divided the patients by the cytosolic Hsp70 protein (major stress-inducible heat shock protein 70) level: patients with a high value of this factor were assigned to cluster 0, and patients with a low value to cluster 0 (Figure 2a). DBSCAN divided the Tainan2020 dataset based on the sex variable: female patients were put in the cluster 0 and male patients in the cluster 0 (Figure 2b). The patients of the Utrecht2019 dataset, instead, were partitioned based on the subventricular zone variable (Figure 2c). We considered the imbalance of each feature on the two clusters as a sign of clear distinction made by DBSCAN for that specific variable  [43].

Glioblastoma exhibits broad biological heterogeneity across tumor-intrinsic stress programs, host determinants, and brain topography. In three independent cohorts (Munich2019, Tainan2020, Utrecht2019), unsupervised density-based clustering (DBSCAN) identified separations dominated by cytosolic heat shock protein 70 (cHsp70), sex, and subventricular zone (SVZ) contact, factors reflecting proteostasis, immune-molecular, and anatomic dimensions of glioblastoma biology that influence treatment tolerance, immune contexture, and recurrence patterns. In the following sections, we explicitly distinguish observations directly supported by DBSCAN clustering from biological interpretations inferred from prior literature.

Heat shock protein 70 is a stress-inducible chaperone protein that maintains proteostasis and suppresses apoptosis, helping glioblastoma cells to survive metabolic stress and therapy  [44]. Depending on its localization, heat shock protein 70 can influence tumor biology and tumor-immune interactions, suggesting that upregulated cytosolic Hsp70 may mark a stress-adapted, glioblastoma phenotype  [44]. In human glioblastoma cohorts, circulating heat shock protein 70 is consistently higher than in healthy controls, supporting its use as a blood-based disease biomarker  [45]. In a large cohort of grade 3–4 gliomas, high circulating Hsp70 with reduced CD3+/CD4+ T-cell frequencies was linked to poorer survival in patients with glioblastoma, whereas higher vesicular heat shock protein 70 with increased activated natural killer (NK) cells correlated with better survival in patients with grade 3 gliomas  [45].

Similarly, an independent study reported that lower serum Hsp70 levels were associated with better overall survival, implying that elevated heat shock protein 70 indicates an unfavorable prognosis  [44]. At the tissue level, cytosolic Hsp70 measured by immunohistochemistry (IHC) in resected glioblastoma showed the opposite pattern: higher intratumoral cytosolic heat shock protein 70 correlated with longer progression-free survival (PFS) and overall survival (OS) under Stupp chemoradiation, most prominently in MGMT-methylated tumors  [12]. Mechanistically, membrane-associated heat shock protein 70 (mHsp70) is present on viable glioblastoma cells, correlates with greater motility/invasion, and its pharmacologic inhibition (PES, JG-98) reduces invasion in patient-derived glioblastoma cells  [46]. These seemingly divergent findings highlight the context-dependent role of Hsp70, whose prognostic effect differs between intracellular expression (favorable under therapy) and extracellular release (unfavorable with tumor burden)  [12, 45]. Accordingly, it is biologically plausible that cytosolic heat shock protein 70 emerged as the discriminant feature in the Munich2019 dataset, clustering patients by stress-response phenotype.

In the Munich2019 dataset, DBSCAN divided patients primarily on the basis of cytosolic Hsp70 levels. Cluster 1 was enriched for cHsp70-high tumors, whereas cluster 0 included mostly cHsp70-low cases. This separation highlights the role of heat shock protein 70 as a stress-response axis within glioblastoma, with clusters reflecting differential tumor capacity to buffer proteotoxic and metabolic stress  [44, 45]. Interestingly, patients in the cHsp70-high cluster appeared to trend toward longer progression-free survival and overall survival, consistent with findings from resected glioblastoma tissues where higher intratumoral cytosolic Hsp70 correlated with improved outcomes under chemoradiation  [12]. The fact that cytosolic heat shock protein 70 emerged as the dominant clustering feature suggests that proteostasis biology is a fundamental organizing principle in this cohort  [12]. One interpretation is that elevated cytosolic Hsp70 reflects tumors that are stress-adapted at the cellular level, while the co-expression of membrane-associated heat shock protein 70 generates stronger immune recognition through stress protein exposure, especially in the context of MGMT promoter methylation and effective chemoradiation  [12, 44]. Conversely, low cytosolic Hsp70 tumors may lack this dual advantage, progressing earlier despite lower intrinsic stress buffering  [12]. Overall, the Munich2019 clustering result supports the idea that the impact of heat shock protein 70 depends on its context. In this cohort, tumors with higher cytosolic Hsp70 formed a distinct group of patients who tended to have better outcomes, consistent with earlier studies showing that tissue cytosolic heat shock protein 70 can act as a positive prognostic marker in glioblastoma  [12].

The Munich2019 findings suggest that cytosolic Hsp70 could be integrated into the biomarker panel for glioblastoma to refine patient stratification beyond MGMT promoter methylation  [12]. Tissue expression of cytosolic Hsp70 may help identify patients more likely to benefit from standard chemoradiation, while circulating or membrane-associated heat shock protein 70 could support liquid-biopsy approaches and inform immune-targeted strategies  [12, 44, 46]. The overlap between Hsp70 biology and natural killer (NK) cell activity further raises the possibility that heat shock protein 70 status might guide the design of immunotherapy combinations  [44, 46]. These implications underscore the translational potential of Hsp70 as both a prognostic and therapeutic marker in glioblastoma. If validated prospectively, tumors with high cytosolic heat shock protein 70 could mark patients likely to derive maximal benefit from standard chemoradiation and who might be candidates for trials combining Hsp70‑directed or natural killer cell augmented approaches  [12, 44].

Sex is a fundamental biological variable influencing cancer incidence, molecular pathways, and treatment response. In glioblastoma, recent multi-omics and immunologic studies reveal distinct male-female differences in clinical and biological characteristics  [47–49]. Females with glioblastoma more frequently show MGMT promoter methylation and other distinct molecular traits, whereas males with glioblastoma are enriched for EGFR-RTK signaling and exhibit greater T-cell exhaustion, providing a plausible biological basis for sex-associated clustering  [47, 49].

Notably, the following interpretations draw on external molecular and immunologic studies, as such pathway-level variables were not directly encoded in Tainan2020.

Multi-omics analyses show clear molecular divergence by sex  [47]. In glioblastoma, tumors from male patients are enriched for EGFR-RTK signaling (including increased EGFR phosphorylation) with sex-specific prognostic associations, whereas tumors from female patients display distinct programs (for example, SPP1/osteopontin axis) and higher frequencies of MGMT-promoter methylation reported in other cohorts  [47, 50]. Sex also shapes the immune microenvironment. Glioblastoma tumors from male patients show greater T-cell exhaustion (for example, PD-1/TOX high) and weaker cytokine production, while female T cells retain stronger cytotoxic/IFN-$\gamma$ programs  [49]. Mechanistically, the X-escape gene KDM6A/UTX supports superior female T-cell effector function  [49]. These immune differences have clinical implications. Meta-analytic data indicate better 1-year survival in females receiving glioblastoma immunotherapies, with a larger effect in vaccine-based approaches  [48]. Clinically, large IDH-wildtype glioblastoma cohorts show higher MGMT promoter methylation and older age at diagnosis in women, but survival differences vary by dataset and adjustment model, reflecting the multifactorial, context-dependent nature of sex effects  [50]. Other data corroborates this, with one 464-patient cohort reporting MGMT methylation in 52% of females vs. 37% of males. Critically, that study also found the survival benefit from MGMT methylation was significant in females, highlighting a key sex-specific treatment interaction  [51]. Taken together, contemporary molecular, immune, and radiologic evidence supports sex as a dominant separating axis in glioblastoma (as seen in the Tainan2020 DBSCAN clusters), even without a consistent survival difference across settings.

In the Tainan2020 dataset, DBSCAN primarily separated patients by sex, with cluster 0 containing females and Cluster 1 containing males. Other variables showed minor between-cluster shifts. This pattern is biologically plausible given recent multi-omics and immunology data. Male glioblastomas show preferential activation of EGFR-RTK signaling, which is linked to proliferative programs, and greater T-cell exhaustion, a state associated with diminished antitumor immunity  [47, 49]. By contrast, female glioblastomas more often exhibit MGMT-promoter methylation and DNA-repair related programs, features that can shape therapeutic sensitivity and overall disease biology. Large clinico-radiogenomic data also corroborate higher MGMT-methylation frequency in women  [47, 50]. Taken together, the Tainan2020 split appears to capture a sex-linked biological axis; the male-enriched cluster is consistent with EGFR/PI3K-skewed, more immunologically exhausted tumors reported in males, whereas the cluster contain females is consistent with a repair/MGMT-enriched state, rather than differences in treatment delivery recorded in this dataset. Accordingly, this mapping is hypothesis-generating and should be validated in external cohorts with integrated molecular and immune profiling.

Sex emerged as a dominant separating axis in the Tainan2020 cohort and is therefore presented here as an empirical clustering result rather than a prespecified variable.

Consistent with best practice, sex-associated clustering should be interpreted in a stratified manner, acknowledging that sex may act as a proxy for underlying molecular, immune, hormonal, or treatment-response differences that were not directly encoded in this dataset. Residual confounding by unmeasured variables cannot be excluded, and the present findings should therefore be interpreted cautiously. Within this context, future studies should pre-specify sex-stratified analyses and incorporate correlative molecular and immune endpoints  [47].

Immunotherapy trials should include sex-stratified immune profiling, as males with glioblastoma exhibit greater T-cell exhaustion and females demonstrate a modest survival advantage with immunotherapies  [48, 49]. In prognostic models, sex should be considered alongside MGMT: large IDH-wildtype cohorts report higher MGMT-promoter methylation in women but no independent sex effect on survival after adjustment, supporting its role as a stratifier rather than a stand-alone prognostic variable  [50].

The subventricular zone (SVZ), an adult neural stem-cell niche along the lateral ventricles, harbors stem cells implicated in glioblastoma initiation and recurrence, with mutated subventricular zone neural stem cells (NSCs) potentially driving tumor regrowth after resection  [52, 53]. Clinically, glioblastomas that contact the subventricular zone at diagnosis show worse outcomes. Subventricular zone contact is an independent negative prognostic factor for survival, and closer proximity associates with shorter progression-free survival  [54, 55].

Subventricular zone contact is a robust prognostic signal. In a glioblastoma cohort, tumors contacting the subventricular zone were larger, had more MGMT-unmethylated cases, and showed shorter overall survival and progression free survival  [54]. In multivariable analysis, subventricular zone contact remained an independent adverse factor  [54]. Beyond a binary label, proximity to the subventricular zone also matters. In a clinicogenetic cohort, subventricular zone contact predicted poorer progression-free survival after adjustment, and each incremental increase in distance from the subventricular zone was associated with longer progression-free survival; overall survival showed a borderline trend  [55]. Mechanistically, the subventricular zone behaves as a neural stem-cell niche relevant to recurrence biology. Deep sequencing across longitudinal tissues found genetic links between recurrent tumors and the subventricular zone in 60% of patients, supporting a model in which mutation-harboring subventricular zone neural stem-cells can seed regrowth toward the resection cavity (via CXCR4–CXCL12 signaling)  [53]. Therapeutically, while the subventricular zone is a biologically attractive target, dose escalation to the subventricular zone has not consistently improved survival. In a trimodal cohort, higher mean dose to ipsilateral/contralateral SVZ did not correlate with better overall survival/progression-free survival on multivariable analysis; patients whose tumors directly involved the subventricular zone had worse overall survival  [52].

Within the constraints of binary subventricular zone encoding in the Utrecht2019 dataset, DBSCAN identified subventricular zone contact as the dominant separating variable, with other recorded variables showing comparatively smaller between-cluster shifts in this cohort. In the Utrecht2019 dataset, DBSCAN primarily separated patients by subventricular zone contact, with other recorded variables showing comparatively smaller between-cluster shifts in this cohort.

External studies show that subventricular zone-contacting glioblastomas present with larger volumes and more MGMT-unmethylated cases and have independently shorter overall survival and progression free survival. Moreover, greater distance from the subventricular zone is associated with longer progression-free survival This axis is clinically meaningful, aligning with external evidence linking subventricular zone contact to larger tumor volume, MGMT-unmethylated status, and independently shorter survival  [54, 55]. Biologically, the subventricular zone functions as a neural stem-cell niche that can harbor driver-mutant neural stem cells genetically linked to recurrent tumors after resection, offering a plausible mechanism for why subventricular zone involvement emerges as a dominant separating feature  [53].

Because Utrecht2019 encodes subventricular zone contact as a binary variable and lacks pathway, immune, or recurrence-pattern data, the observed separation should be regarded as hypothesis-generating. Although clustering quality was high, only 24% of cases formed clusters, underscoring the need for prospective, multi-centre validation with integrated molecular and immune profiling and quantitative subventricular zone metrics (for example, distance and extent of contact) to determine whether subventricular zone involvement defines a biologically distinct subset and how it should inform risk stratification. Clinically, SVZ-contacting glioblastomas may warrant enhanced surveillance for leptomeningeal or distant spread and the use of quantitative SVZ metrics in reports. Whether elective SVZ coverage or boosting improves outcomes remains unsettled and should be framed cautiously in light of consensus guidance  [56].

Across three independent cohorts, unsupervised clustering converged on biologically coherent axes that organize glioblastoma beyond routine clinical variables: a proteostasis/stress program marked by cytosolic heat shock protein 70, sex-linked molecular and immune divergence, and ventricular niche involvement via subventricular zone contact. In Munich2019, higher cytosolic Hsp70 aligned with a stress-adapted, therapy-responsive tissue phenotype; in Tainan2020, sex captured established differences in EGFR/PI3K signaling, MGMT/repair programs, and T-cell exhaustion; in Utrecht2019, subventricular zone contact reflected a stem-cell-niche-associated pattern with recognized prognostic and spatial implications.

Taken together, these findings suggest that cytosolic heat shock protein 70, sex, and subventricular zone contact alongside MGMT and other standards can be considered hypothetical key elements for glioblastoma, to be prospectively further tested in the future.

Because pathway, immune, and spatial metrics were not uniformly available, our interpretations are hypothesis-generating and warrant external, multicenter validation with integrated molecular/immune profiling and quantitative subventricular zone measures to determine clinical utility and incorporate these axes into risk stratification.

### Explainability

We also performed a feature ranking analysis on the three datasets, to understand which variables were contributing more to the final DBSCAN clusters. We compute the permutation importance of each dataset feature on the DBSCAN clustering results by fitting DBSCAN on the original data, permuting one feature at a time, re-executing DBSCAN, and measuring how different the new clustering is from the original using the Adjusted Rand Index (ARI). The bigger the change is, the more important the feature is. We report the results of this analysis in Table 4, which clearly indicate that adjuvant treatment made a huge contribution to the DBSCAN clusters on the Utrecht2019 dataset, survival particularly impacted the DBSCAN results on the Munich2019 dataset, and diagnostic year was the most influencing variable on the DBSCAN results of the Tainan2020 dataset.

**Table 4:** Feature importance values

Utrecht2019		Munich2019		Tainan2020	
**feature**	importance	feature	importance	feature	importance
1 adjuvant_treatment	0.838037	survival	0.588803	diagnostic year	0.440017
2 biopsy0_resection1	0.651395	MGMT_methylation	0.580813	surgery	0.438412
3 KPS_greater_or_equal70	0.527367	sex_0f_1m	0.521667	Chemogroup	0.391662
4 alive0_deceased1	0.341252	PFS_months	0.478355	TMZ	0.379161
5 survival_days	0.241737	cHsp70_0low_1high	0.374614	sex_1male_0female	0.220121
6 age	0.203596	age_years	0.309477	Chemo	0.150121
7 SVZ_contact	0.130313	did_progress	0.119481	volume_mL	0.132322
8				statusOS	0.108680
9				dose	0.097574
10				age_years	0.082839
11				PFS	0.075544
12				age	0.071190
13				OS	0.069100
14				statusPFS	0.010555

Importance: adjusted Rand index drop when that feature is removed, with respect to the adjusted Rand index value when all the features are used. Adjusted Rand index range: $[-1,+1]$, the higher the better

### Cluster robustness and stability

To verify the robustness and the stability of the clusters identified by DBSCAN, we performed a bootstrap analysis  [57] with ten resamplings, which produced the following average plus-minus standard deviation results for the DBCV index, in the $[-1,+1]$ interval:

- Munich2019: +0.3255 $$\pm$$ 0.1140
- Tainan2019: +0.2805 $$\pm$$ 0.0675
- Utrecht2020: +0.2131 $$\pm$$ 0.0447

As one can notice, these results are profoundly different from the results obtained on the DBSCAN results without bootstrap and without subsampling (Table 3). This outcome is probably due to the low dimensionality of the datasets (Table 1) and to the non-determinism of the DBCV index  [58].

## Discussion and conclusions

The current study has several assets. To the best of our knowledge, our project is the first clustering study using open medical data records of patients with glioblastoma and employing only open source software libraries. We found no other article describing the application of a fully-unsupervised approach data of this particular brain tumor.

Our results demonstrate that DBSCAN clustering, paired with the DBCV index, can identify groups of patients with significant medical traits among data derived from electronic health records. Moreover, our results indicate that some clinical features can be more useful than others to partition the data of patients in a medically significant way: cytosolic Hsp70 protein for the Munich2019 dataset, sex for the Tainan2020 dataset, and brain subventricular zone for the Utrecht2019 dataset. Each of these three clinical factors is known to have a significant role in glioblastoma  [59–61]. These promising preliminary results appear to proof the capability of DBSCAN and DBCV to identify clusters of patients that have a medical meaning, paving the way to further analyses.

It is also important to note that our results might be influenced by biases in the dataset, which can stem from demographic, clinical, or data-driven characteristics of the patient samples. This means that our findings, even if valid for the analyzed datasets, may not be generalizable to all possible cohorts of patients diagnosed with glioblastoma. For these reasons, our discoveries regarding cytosolic Hsp70 protein, sex, and subventricular zone should be regarded as initial hypotheses or starting points for further research, rather than definitive conclusions.

Our data-driven approach identified these three clinical-biological variables as the most relevant for cluster discrimination. However, readers should not assume that these three attributes are overwhelmingly more important than other variables related to treatment or outcomes. Cytosolic Hsp70 protein, sex, and the subventricular zone are highlighted in this study, but their significance should not be overinterpreted or overestimated.

In any case, this study represents an indicative case of responsible use of machine learning in healthcare: the algorithm and the metric employed (DBSCAN and DBCV index), in fact, can be clearly interpreted and explained to anyone, even without a deep knowledge on machine learning. Our approach can be be considered fair because our clustering methods do not produce biased outcomes based on sensitive attributes such as race, sex, gender, or socioeconomic status. Moreover, the privacy of patients is preserved by the anonymity of data: nobody can trace the identity of patients from the data, even in the remote case they wanted to. The datasets were collected by the original data curators after obtaining the informed consents from the patients and the authorization of the ethical committees of the corresponding hospitals  [12–15]. The anonymous datasets were then released openly following the FAIR principles  [16]. Regarding explainability and interpretability, we decided to use a modern clustering algorithm (DBSCAN) whose functioning is known and can be explained to anyone. DBSCAN, in fact, is not a black-box model  [62]. Our project is fully patient-centric: our primary goal was on improving patient outcomes, and we did it by identifying the main clinical features that can discriminate the clusters of patients in the three analyzed datasets. Of course, these practices can be generalized to any biomedical informatics research project.

Among the three highlighted features from electronic medical records, only sex can be considered an early-available variable suitable for patient stratification and the design of more effective therapies. Unfortunately, cytosolic Hsp70 protein levels and the presence of contact between glioblastoma and the brain subventricular zone are not immediately available for patients diagnosed with this cancer type. Clinicians could leverage our findings by giving greater consideration to the patient’s sex when assessing prognosis.

Regarding limitations, we must note that performing a cluster stability analysis and obtaining stronger external validation would further contextualize the findings from the three datasets we examined. Additionally, our analysis relied on a single metric (the DBCV index), whereas it is generally advisable to use multiple evaluation coefficients in machine learning studies. Two of the three datasets (Munich2019 and Tainan2020) have relatively small sample sizes, with 60 and 84 patients respectively, which clearly impacted the clustering results. Moreover, the variables across the three datasets are highly heterogeneous. It would have been preferable to have larger datasets consisting primarily of similar data types.

Our clinical considerations on the clustering results should be seen as a focus suggestion for medical doctors, and no clinical decision-making should be based on these clusters to date. Additionally, the observed cluster differences by sex in the Tainan2020 dataset should not be taken at face value. Since scientific evidence about sex as a glioblastoma predictor is mixed and since MGMT status and patients treatment might influence or confuse the results, these sex-based patterns need careful interpretation considering these complexities. In the future, we plan to analyze the relationship between clusters and outcomes, in the datasets where this information is available. We also plan to enhance clustering by applying dimensionality reduction [63, 64] beforehand to see if the results improve. Moreover, we will test whether these unsupervised clusters can predict survival or treatment response in external validation cohorts with harmonized variables.

## Data Availability

No datasets were generated or analysed during the current study.

## Software availability

Our Python scripts can be openly found on Google Colab at https://colab.research.google.com/drive/1hyE58egraDylwyHjKbvg-wfJnGTzRbEv?usp=sharing

## Abbreviations

BIRCH: Balanced iterative reducing and clustering using hierarchies
cHsp70: Cytosolic heat shock protein 70
CRAN: Comprehensive R Archive Network
CXCL12: C-X-C motif chemokine ligand 12
CXCR4: C-X-C chemokine receptor type 4
DBCV: Density-Based Clustering Validation
DB392 SCAN: Density-based spatial clustering of applications with noise
EGFR: Epidermal growth factor receptor
FAIR: Findability, accessibility, interoperability, and reusability
HDBSCAN: Hierarchical density-based spatial 394 clustering of applications with noise
Hsp70: Heat shock protein 70
IDH: Isocitrate dehydrogenase
IFN-$\gamma$: Interferon gamma
IHC: Immunohistochemistry
JG-98: Small-molecule Hsp70 inhibitor
KDM6A/UTX: Lysine demethylase 6A (ubiquitously transcribed tetratricopeptide repeat. X chromosome)
KPS: Karnofsky 397 Performance Scale
mHsp70: Membrane-associated heat shock protein 70
mL: Milliliters
MGMT: Methylated- 398 DNA–protein-cysteine methyltransferase
NK: Natural killer
OS: Overall survival
OPTICS: Ordering Points 399 To Identify the Clustering Structure
PD-1: Programmed cell death protein 1
PES: 2-phenylethynesulfonamide
PFS: Progression-free survival
PI3K: Phosphoinositide 3-kinase
RTK: Receptor tyrosine kinase
SPP1: Secreted phosphoprotein 1
SVZ: Sub ventricular zone
TCGA: The Cancer Genome Atlas
TOX: Thymocyte 402 selection-associated high mobility group box protein
